# Organizing Pneumonia As the Initial Manifestation of Systemic Lupus Erythematosus: Case Report and Literature Review

**DOI:** 10.7759/cureus.108036

**Published:** 2026-04-30

**Authors:** Leidy P Cespedes Useche, Juan Manuel Bello, Daniel Celis Giraldo, Deider S García Villamizar

**Affiliations:** 1 Internal Medicine, Central Military Hospital, Nueva Granada Military University, Bogotá D.C., COL; 2 Rheumatology, Central Military Hospital, Bogotá D.C., COL

**Keywords:** case report, initial manifestation, interstitial lung disease, organizing pneumonia, systemic lupus erythematosus

## Abstract

Systemic lupus erythematosus (SLE) can affect multiple organs, including the lungs, with a reported frequency ranging from 20% to 90%. Organizing pneumonia (OP) is a rare and uncommon presentation that may be life-threatening.

A 42-year-old male presented with respiratory symptoms, fever, a history of recurrent pneumonia, and the development of oral ulcers. Antinuclear antibodies were positive with an AC-01 pattern, and he scored 10 points according to the 2022 European Alliance of Associations for Rheumatology (EULAR) classification criteria, establishing the diagnosis of SLE. Corticosteroid therapy was initiated with clinical resolution. We conducted a search in the PubMed database for cases of systemic lupus erythematosus that initially presented with organizing pneumonia. The search terms used were “organization” or “organized”, or “organizing”, and “pneumonia” and “lupus” or “systemic lupus erythematosus”. It should be noted that no time filter was applied, and reports of cases of individuals under 18 years of age were excluded.

OP was diagnosed as the initial manifestation of SLE, representing a diagnostic challenge in the absence of other guiding symptoms. We identified 19 case reports describing OP as the initial presentation of SLE.

This case illustrates the diagnostic complexity and variability of SLE, and how OP, in the appropriate clinical context, should raise suspicion of SLE onset.

## Introduction

Interstitial lung disease (ILD) encompasses a heterogeneous group of over 200 disorders, each with variable clinical presentations and prognoses. Due to the progressive nature of most ILDs, early diagnosis and intervention are crucial to improve prognosis and survival [1]. In the United States, the estimated prevalence of ILD is approximately 199 per 100,000 individuals, with a higher prevalence observed in women [2]. ILDs are classified into five categories based on etiology: primary associated diseases (sarcoidosis, eosinophilic pneumonia, lymphangioleiomyomatosis, or primary alveolar proteinosis), environmentally related exposures (pneumoconiosis and chronic hypersensitivity pneumonitis), drug-induced, chemical, or radiation-related ILDs, connective tissue disease-associated ILDs (CTD-ILDs) (including rheumatoid arthritis, systemic sclerosis, Sjögren's syndrome, inflammatory myopathies, anti-synthetase syndrome, systemic lupus erythematosus, and mixed connective tissue disease), and idiopathic interstitial pneumonias, which include idiopathic pulmonary fibrosis and nonspecific interstitial pneumonia (NSIP) [3-5]. CTD-ILD accounts for approximately 25% of ILDs and is the second most common subtype [4,6]; thus, investigating CTD in patients presenting with ILD is of great importance.

Most patients with CTD-ILD initially develop respiratory symptoms secondary to ILD after their CTD diagnosis. However, a small proportion of patients present with ILD as the initial manifestation of an underlying connective tissue disease [2,6]. Histopathologically, the spectrum of ILD includes NSIP, usual interstitial pneumonia (UIP), organizing pneumonia (OP), apical fibrosis, diffuse alveolar damage, and lymphoid interstitial pneumonia, with NSIP being the most commonly encountered pattern. UIP is considered rare and remains infrequently reported, according to a systematic review and meta-analysis published in 2023, and typically occurs in the context of a pre-existing CTD diagnosis [7-9]. In systemic lupus erythematosus (SLE), approximately 3-8% of cases present with clinically significant ILD; among the various pathological and radiological patterns, NSIP is the most frequently observed [10]. Given its low frequency and the diagnostic challenge it poses initially, mainly due to its potential reversibility with early treatment, we present a case of SLE in which organizing pneumonia (OP) was the initial manifestation.

## Case presentation

This is a 42-year-old male patient from Bogotá D.C., Colombia, a former military personnel, who presented with a three-day history of cough, mucopurulent expectoration, fever, chills, rhinorrhea, general malaise, asthenia, and self-limited diarrhea. Past medical history included type 2 diabetes mellitus (DM2), obstructive sleep apnea (OSA), asthma, and community-acquired pneumonia (CAP) seven months prior, treated with ampicillin/sulbactam plus clarithromycin.

During systemic review, recurrent nasal congestion, painful oral ulcers, malar region desquamation, and erythematous nodules on the lower limbs that resolved spontaneously were noted. In the previous hospitalization for CAP, transient hypereosinophilia (3,970/mm³) was documented. On physical examination, vital signs were stable; auscultation revealed decreased vesicular breath sounds at the lung bases and crackles at the right base. Laboratory tests on admission showed a normal complete blood count, elevated C-reactive protein (Table 1), and a negative severe acute respiratory syndrome coronavirus 2 (SARS-CoV-2) antigen test. Chest X-ray revealed right basal alveolar consolidation and right perihilar atelectasis, leading to a presumptive diagnosis of recurrent pneumonia.

**Table 1:** Laboratory results

Test Name	Result	Reference value
C-reactive protein	8.84 mg/dL	0 - 0.5 mg/dL
Antinuclear antibodies (ANA)	1:640 homogeneous pattern	Positive: > 1/80
Anti-cardiolipin IgG	8.3 GPL	Negative: < 15 GPL
Anti-cardiolipin IgM	33.59 MPL	Negative: < 12.5 MPL
Anti-beta-2 glycoprotein IgG	3.43 U/mL	Negative: < 20 SGU
Anti-beta-2 glycoprotein IgM	5.99 U/mL	Negative: < 20 SMU
Anti-dsDNA antibodies	60.8 IU/mL	Negative: 0-200 UI/mL
Anti-Smith (SM) antibodies	2.8 U/mL	Negative: < 20 U/mL
Complement C3	66.9 mg/dL	81.1 - 157 mg/dL
Complement C4	1.7 mg/dL	12.9 – 39.2 mg/dL

The patient was started on empirical antibiotic therapy with ampicillin/sulbactam and clarithromycin. Further workup included a GeneXpert Mycobacterium tuberculosis (MTB)/ Rifampicin (RIF) assay (Cepheid Inc., Sunnyvale, USA) in sputum (negative), cultures for common bacteria (negative), and a multiplex polymerase chain reaction (PCR) panel for pneumonia pathogens (negative). HIV testing by Enzyme-Linked Immunosorbent Assay (ELISA) was also negative.

**Figure 1:**
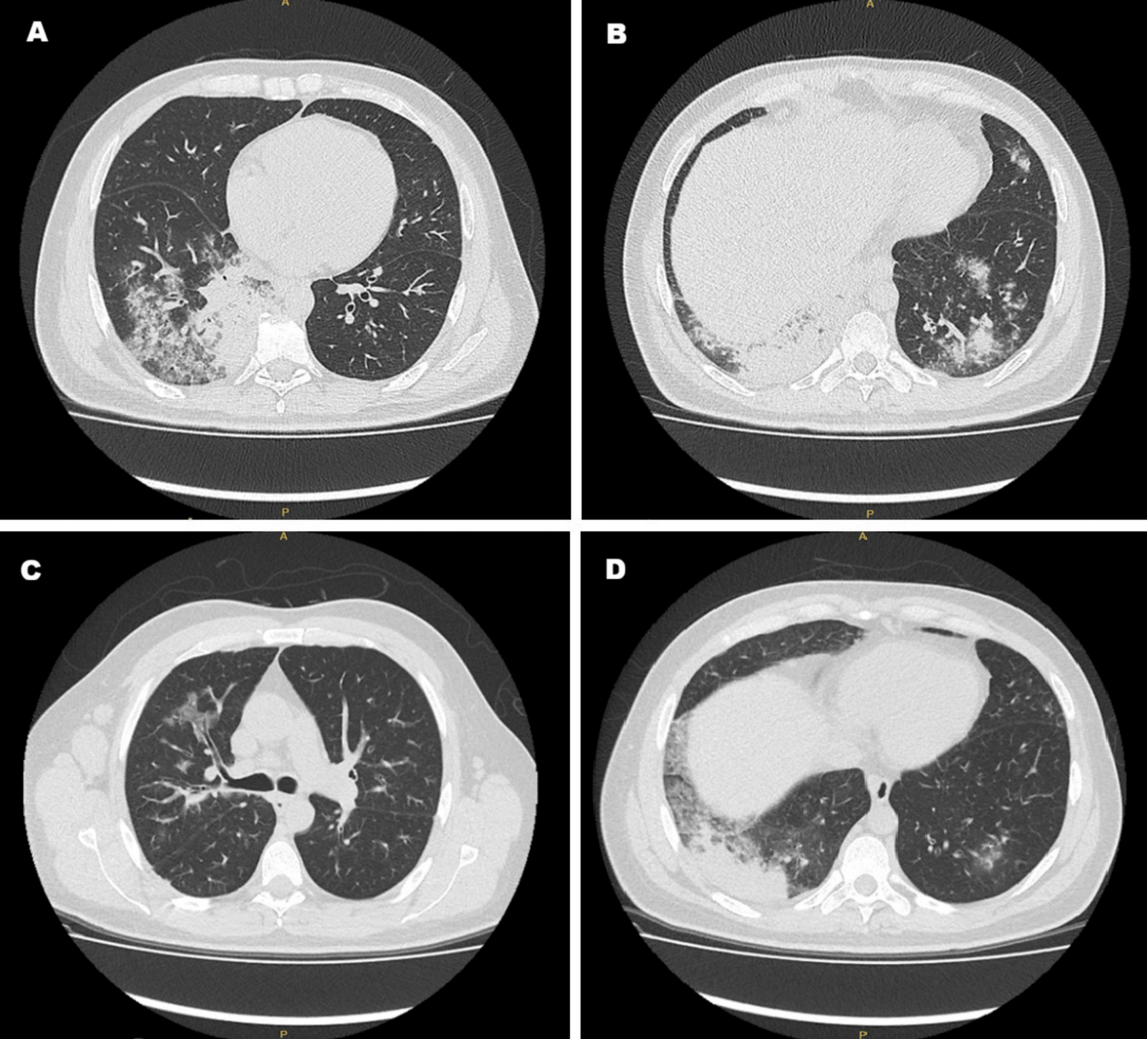
High-resolution computed tomography (HRCT) of the chest during the first (A and B) and second episode (C and D) A and B: An alveolar occupation pattern with consolidative appearance is observed, associated with air bronchogram configuration and peripheral ground-glass opacities at the level of the posterobasal segments of both hemithoraces, with greater involvement of the right lung. C and D: Multiple areas of ground-glass opacities are identified in the right upper lobe and right lower lobe. A posterior basal right parenchymal consolidation with peripheral ground-glass opacities and air bronchogram is noted. Incipient ground-glass areas are also seen in the left lung.

High-resolution computed tomography (HRCT) of the chest demonstrated ground-glass opacities and migrating consolidations compared to previous imaging (Figure 1). Bronchoscopy with bronchoalveolar lavage (BAL) and transbronchial biopsy were subsequently performed.

**Figure 2:**
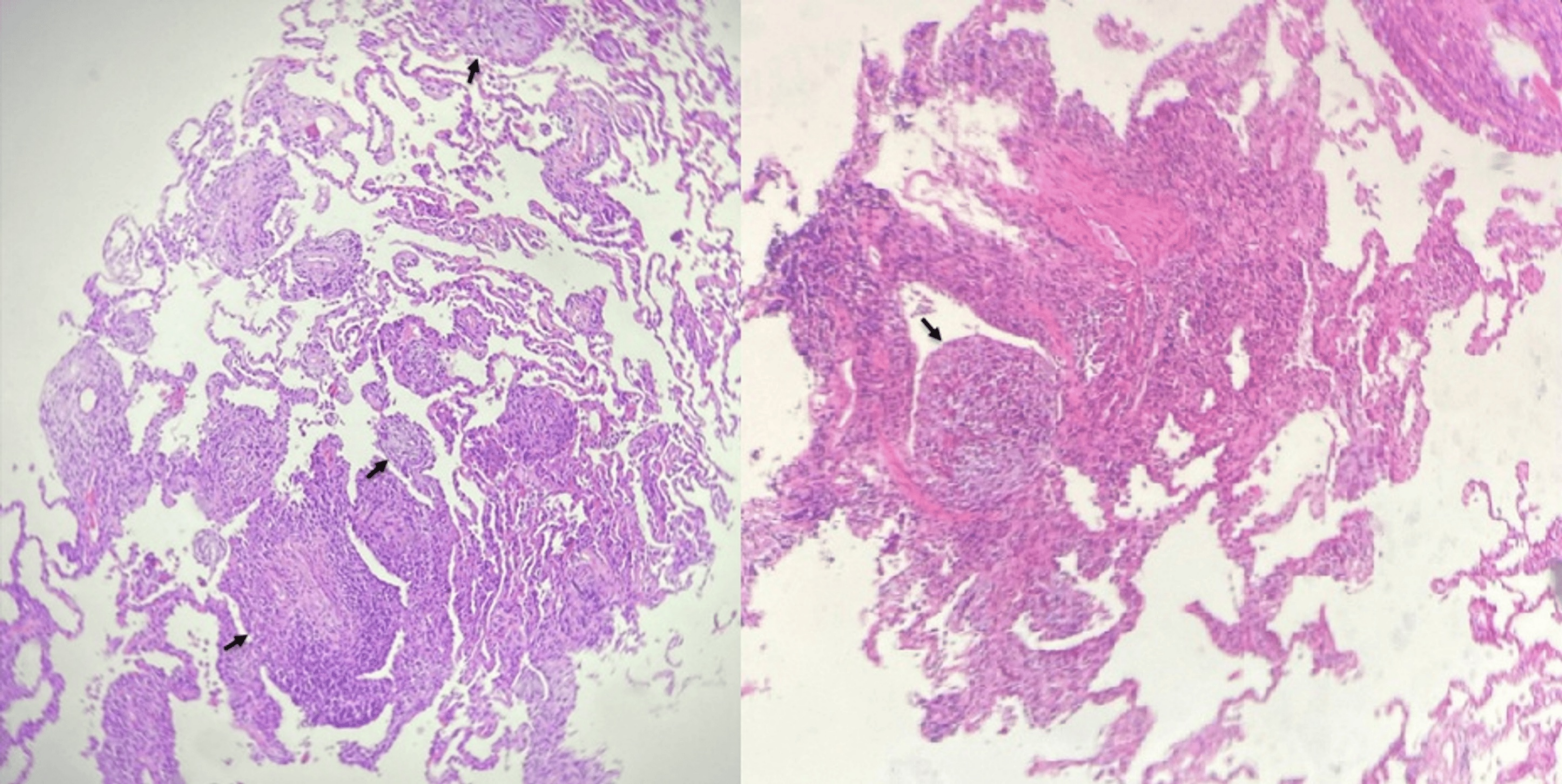
Hematoxylin and eosin (H&E) staining of tissue from the right lower lobe transbronchial biopsy, lateral segment: The bronchial mucosa is lined by respiratory epithelium without atypia. Alveolar septa are slightly thickened with a mild to moderate lymphoplasmacytic infiltrate and some neutrophils. The epithelium is covered by reactive type II pneumocytes with fibroblastic polyps (Masson bodies; black arrows). Alveolar spaces contain histiocytes. Eosinophilic infiltrate is minimal (2 eosinophils per 10 high-power fields). No granulomas, fibrosis, vasculitis, or tumor are observed.

**Figure 3:**
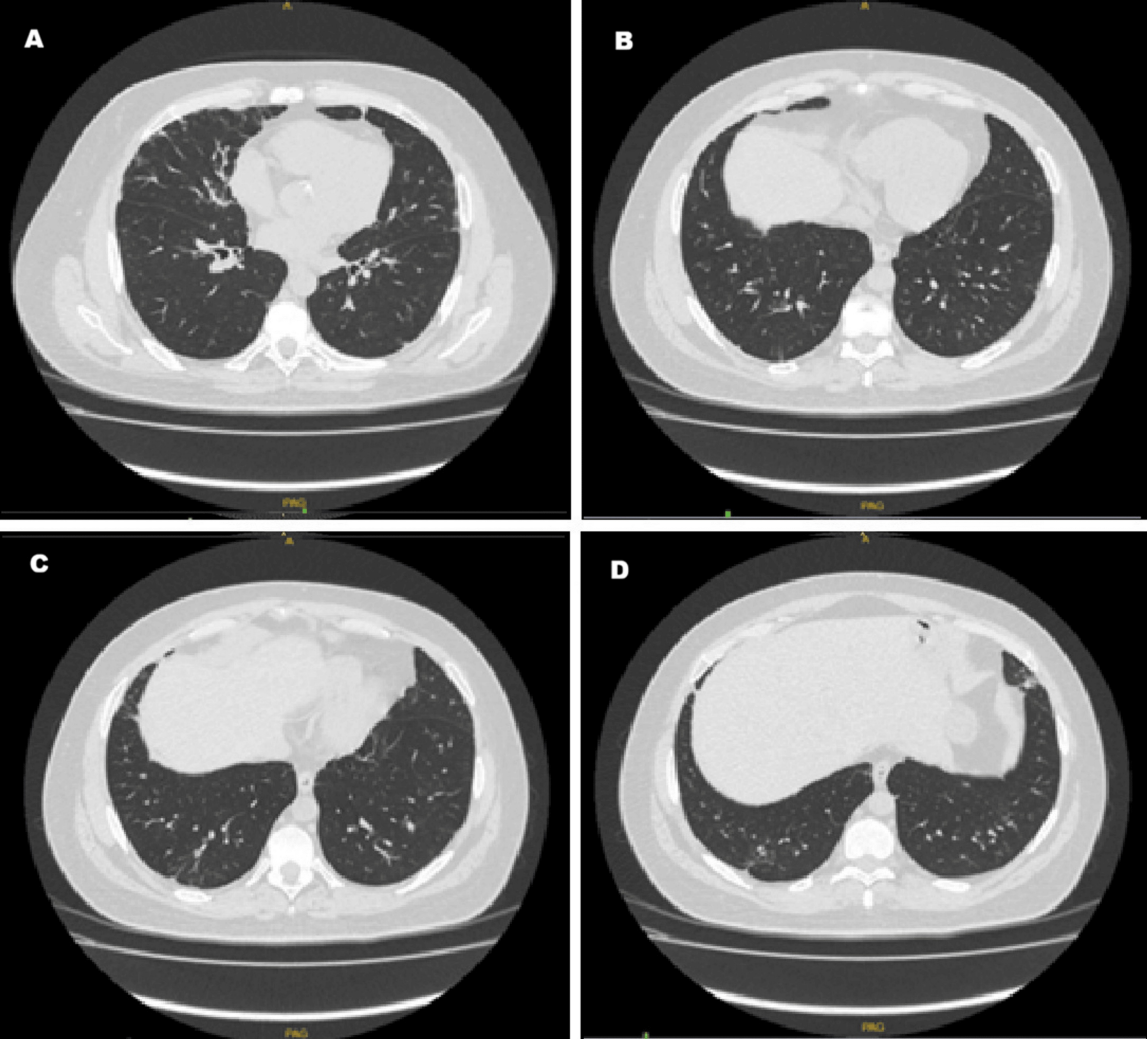
High-resolution computed tomography (HRCT) of the chest, performed five months after initiation of treatment A - D: Resolution of alveolar opacities is observed

Throughout follow-up, the patient presented with chronic nasal congestion. A CT scan of the paranasal sinuses revealed chronic pansinusitis with inflammatory changes. A diagnosis of eosinophilic granulomatosis with polyangiitis (EGPA) was suspected due to prior eosinophilia, chronic pansinusitis, late-onset asthma, and pulmonary involvement. An antineutrophil cytoplasmic antibody (ANCA) test was performed, which was negative, thereby making EGPA less likely. Considering the presence of oral ulcers, connective tissue disease, specifically systemic lupus erythematosus (SLE), was also suspected. The bronchoalveolar lavage (BAL) report was negative for infections, including fungal, and showed lymphocytosis (20%). After seven days of antibiotic therapy, the patient showed clinical improvement and was discharged. One month later, he re-presented with respiratory symptoms, including a cough associated with dyspnea. Hospitalization was extended to continue diagnostic assessments. A previous transbronchial biopsy had demonstrated organizing pneumonia (OP), as shown in Figure 2. Serological testing revealed positive antinuclear antibodies (ANA) with a homogeneous pattern. The antibody profile included negative anti-cardiolipin IgG, positive anti-cardiolipin IgM, negative anti-beta-2 glycoprotein IgG, negative anti-beta-2 glycoprotein IgM, negative anti-dsDNA antibodies, negative anti-Smith (SM) antibodies, reduced complement C3 and complement C4 (Table 1). Based on the fulfillment of the classification criteria for SLE (constitutional: fever; mucocutaneous: oral ulcers; antiphospholipid antibodies: anticardiolipin antibodies positive; complement proteins: Low C3 and C4), treatment with antimalarials and systemic corticosteroids was initiated, leading to progressive clinical improvement and radiological resolution, as evidenced by thoracic imaging shown in Figure 3.

## Discussion

A total of 25 cases of organizing pneumonia (OP) associated with systemic lupus erythematosus (SLE) have been reported in the literature, of which 19 presented at disease onset, and six occurred during the disease course as relapses. The presented case represents the 20th reported instance where OP was the initial manifestation of SLE [11-15]. The main characteristics of the reported cases are summarized in Table 2. OP is a pattern of lung tissue repair following injury, which can be cryptogenic (idiopathic) or a response to a specific pulmonary insult. It is of significant clinical interest because it is frequently misdiagnosed, yet appropriate treatment is associated with a high rate of recovery. Secondary OP is attributable to a specific cause such as viral infection, drug toxicity, inhalational injury, radiotherapy, or malignancy, and occurs in the context of a defined clinical setting, including connective tissue diseases, aspiration, transplant sequelae, or other interstitial pneumonias [16]. Histopathologically, OP is characterized by granulation tissue within the alveoli, alveolar ducts, and bronchiolar lumens, often associated with variable interstitial and airway cell infiltration by mononuclear cells. In addition to clinical and immunological considerations, radiological findings are relevant; HRCT tends to show bilateral irregular infiltrates without honeycombing or reticular pattern, which helps differentiate OP from other forms of chronic interstitial pneumonia [17].

**Table 2:** Comparative analysis of studies on OP as a manifestation of SLE SLE: systemic lupus erythematosus; OP: organizing pneumonia; BOOP: bronchiolitis obliterans organizing pneumonia, IVIG: immunoglobulin G; APS: antiphospholipid syndrome.

Author (Year)	Study Type	Patient Characteristics	OP Presentation	Associated Conditions	Treatment	Outcome	Key Findings
Ito et al. [11]	Case series (3 cases) + literature review	Adults with SLE	OP as a pulmonary manifestation (some initial)	None specified	Corticosteroids ± immunosuppressants	Good response	OP may be an early or concurrent manifestation; responds well to steroids
Taira et al. [12]	Case report	SLE + hemophagocytic syndrome	Acute fibrinous and organizing pneumonia	Hemophagocytic syndrome	High-dose steroids, IVIG, plasma exchange, cyclophosphamide	Favorable	Severe inflammatory phenotype; requires aggressive immunosuppression
Acharya et al. [13]	Case report	34-year-old male	OP is the initial manifestation of SLE	None specified	Corticosteroids + cyclophosphamide	Good outcome, asymptomatic follow-up	Reinforces OP as a rare initial presentation with a good prognosis if treated
Krishnappriya et al. [14]	Case report	SLE + antiphospholipid syndrome	BOOP (variant of OP)	APS	Immunosuppressive therapy	Clinical improvement	OP may coexist with APS and thrombotic features
Hariri et al. [15]	Case report + review	SLE patient	Acute fibrinous and organizing pneumonia	None specified	Corticosteroids ± immunosuppressants	Variable	Highlights histopathological variants and diagnostic complexity
Giménez et al. [24]	Case report	Refractory SLE-associated OP	Persistent OP despite standard therapy	None specified	Belimumab	Excellent response	Biologic therapy may be effective in refractory OP
Jatwani et al. [25]	Case report	Adult SLE patient	BOOP as initial manifestation	None specified	Corticosteroids	Improvement	Early OP may precede full SLE diagnosis
Integrated evidence [11-15]	Review of 25 cases (Case reports and small case series)	Mixed (majority adults)	19 initial presentations, 6 during disease course	May include systemic activity (e.g., APS, hemophagocytic syndrome)	First-line corticosteroids; refractory cases require additional immunosuppression	Generally favorable; some severe cases	OP is a rare but clinically significant pulmonary manifestation of SLE, often misdiagnosed; early recognition improves outcomes

OP is associated with several connective tissue disorders such as rheumatoid arthritis (RA), SLE, Sjögren’s syndrome, dermatomyositis, and polymyositis [18]. Pulmonary involvement in SLE poses a clinical challenge, owing not only to its relatively low prevalence compared to other manifestations but also because of its high morbidity and potential mortality. Some studies report that initial pulmonary manifestations occur in approximately 3% of SLE patients, increasing to 7% during follow-up exceeding 10 years [19, 20]. Despite this low incidence, multiple investigations have demonstrated that pulmonary manifestations in SLE, when present, can be severe and often require intensive therapeutic interventions. Common manifestations include pleuritis or pneumonia, but others involve the parenchyma, blood vessels, and airways. Parenchymal involvement includes rare entities such as acute pneumonitis or OP; the latter being an exceptional initial presentation [21]. OP can also appear during disease reactivation, frequently accompanied by systemic symptoms such as fever, rashes, arthritis, and lupus nephritis, supporting a strong association with systemic disease activity [20]. The majority of patients improve with immunosuppressive therapy, with corticosteroids being the cornerstone of treatment. In refractory cases, the addition of immunosuppressants such as mycophenolate mofetil or cyclophosphamide may be necessary [22]. The need for intensive immunomodulation underscores the potential severity of OP as a manifestation of SLE, with documented cases progressing to respiratory failure and death if treatment is inadequate. Diagnosing OP in SLE is challenging due to overlapping clinical and radiological features with other interstitial lung diseases, such as diffuse alveolar damage or acute fibrosing organizing pneumonia [15].

Cases of bronchiolitis obliterans organizing pneumonia (BOOP) associated with SLE and antiphospholipid syndrome (APS) have been reported, often presenting with dry cough, oral ulcers, and malar erythema, along with recurrent deep vein thrombosis [14]. Other reports describe fibrinous and acute organizing pneumonia in patients with SLE and hemophagocytic syndrome, which responded favorably to high-dose corticosteroids, plasma exchange, intravenous immunoglobulin, and cyclophosphamide [12]. OP is considered an inflammatory condition rather than a fibrotic process [23].

A case involving a 34-year-old male presented with features similar to this report, responding well to corticosteroids and consolidating with cyclophosphamide; he remained asymptomatic at follow-up until publication [13]. The efficacy of corticosteroid therapy remains debated, and it is typically combined with other immunomodulators in cases of SLE-related OP. In some refractory cases, the use of belimumab has been reported with excellent clinical and radiological outcomes [24]. Another case documented a patient who had a history of OP diagnosed eight years before being diagnosed with SLE. The high relapse rate despite adequate initial corticosteroid therapy suggests a causal relationship with underlying SLE, which was likely the primary presenting manifestation at that time. OP was attributed as the initial manifestation of SLE because laboratory analyses from that period showed positive ANA results, and the patient experienced several subsequent hospitalizations for recurrent pleural effusions. The initial diagnostic evaluation was incomplete, and early identification of underlying connective tissue disease could have potentially prevented relapses [25].

In this case, clinical manifestations attributed to SLE appeared within less than a year of disease onset. This highlights the importance of close clinical follow-up and precise correlation between the clinical and radiological.

It is important to know that this report is limited by its single-case design, which restricts generalizability. Additionally, the relatively short follow-up limits the assessment of long-term outcomes and relapse. Finally, the small number of published cases and potential publication bias may affect the interpretation of this association.

## Conclusions

This case underscores the need to consider systemic lupus erythematosus in patients presenting with organizing pneumonia of unclear etiology. Despite its rarity, OP can be the first manifestation of SLE and may mimic infectious or other inflammatory conditions, leading to potential delays in diagnosis. A multidisciplinary approach integrating clinical, radiological, and immunological findings is essential. Early identification and prompt treatment with corticosteroids and immunomodulatory therapy are critical to improving outcomes and reducing morbidity. Greater awareness of this association may facilitate earlier diagnosis and optimize patient care.
